# Evaluation of safety, feasibility and efficacy of intra-ovarian transplantation of autologous adipose derived mesenchymal stromal cells in idiopathic premature ovarian failure patients: non-randomized clinical trial, phase I, first in human

**DOI:** 10.1186/s13048-020-00743-3

**Published:** 2021-01-06

**Authors:** M. Mashayekhi, E. Mirzadeh, Z. Chekini, F. Ahmadi, P. Eftekhari-Yazdi, S. Vesali, T. Madani, N. Aghdami

**Affiliations:** 1 Department of Endocrinology and Female Infertility, Reproductive Biomedicine Research Center, Royan Institute for Reproductive Biomedicine, ACECR, P. O Box: 16635-148, Royan Allay, Eastern Hafez St, Banihashem Sq., Resalat Highway, Tehran, Iran; 2 Department of Regenerative Medicine, Cell Sciences Research Center, Royan Institute for Stem Cell Biology and Technology, ACECR, P. O Box: 16635-148, Shaghayegh Alley, Banihashem Sq., Resalat Highway, Tehran, Iran; 3 Department of Reproductive Imaging, Reproductive Biomedicine Research Center, Royan Institute for Reproductive Biomedicine, ACECR, Tehran, Iran; 4 Department of Embryology, Reproductive Biomedicine Research Center, Royan Institute for Reproductive Biomedicine, ACECR, Tehran, Iran; 5 Department of Diabetes, Obesity and Metabolism, Reproductive Epidemiology Research Center, Royan Institute for Reproductive Biomedicine, ACECR, Tehran, Iran

**Keywords:** Adipose derived mesenchymal stromal cell, FSH, Premature ovarian failure, Return menstruation, Ovarian volume

## Abstract

**Background:**

Premature ovarian failure (POF) is characterized by the loss of ovarian activity before the age of 40 years. Stem cell therapy has the capability to create a regenerative microenvironment and is a proposed treatment for POF-related infertility due to the presence of renewal folliculogenesis and germ cells in the adult ovaries. In this study, we assessed the safety, feasibility, efficacy and dose adjustment of autologous adipose-derived stromal cells (ADSCs) and their ability to improve ovarian function in POF patients.

**Methods:**

This study was a non-randomized clinical trial, phase I. Nine women with a definitive diagnosis of POF were divided into three groups (*n* = 3 per group) that received either 5 × 10^6^, 10 × 10^6^, or 15 × 10^6^ autologous ADSCs suspension transplanted in the one ovary. Participants were followed-up at 24 h after the transplantation, and at 1 and 2 weeks, and 1, 2, 3, 6, and 12 months after the transplantation. The primary objective was to evaluate the safety of ADSCs transplantation. Secondary objectives included the effects of ADSCs transplantation on the resumption of menstruation, hormones level (Follicle-stimulating hormone (FSH) and anti-Müllerian hormone), ovarian function (Antral follicle count and ovary volume by ultrasonography evaluation) as well as dose escalation.

**Results:**

Participants had not shown any early-onset possible side effects and secondary complications during follow-up. The menstruation resumption was observed in four patients which established for several months. In the 15 × 10^6^ group, two POF patients had a return of menstruation second months after the intervention. Two other POF patients in 5 × 10^6^ and 10 × 10^6^ cell groups reported menstruation resumption at 1 month after the intervention. We observed decreased serum FSH levels of less than 25 IU/l in four patients. In two patients in 5 × 10^6^ and 10 × 10^6^ cell groups, serum FSH showed an inconsistent decline during a 1 year follow up after ADSCs transplantation. The ovarian volume, AMH, and AFC were variable during the follow-up and no significant differences between cell groups (*p* > 0.05).

**Conclusions:**

We showed the intra-ovarian embedding of ADSCs is safe and feasible and is associated with an inconsistent decline in serum FSH. This should be further investigated with a large RCT.

**Trial registration:**

NCT02603744, Registered 13 November 2015 - Retrospectively registered, http://www.Clinicaltrials.gov

## Background

Premature ovarian failure (POF) is a heterogeneous clinical syndrome defined by loss of ovarian activity before the age of 40. POF affects 1–2% of women of reproductive age and is influenced by ethnicity [1, 2]. According to the European Society of Human Reproduction and Embryology (ESHRE) guidelines, diagnostic criteria for POF include oligo/amenorrhea for at least 4 months and an elevated FSH level of > 25 IU/l on two occasions greater than 4 weeks apart [3]. Potential etiologies for POF are diverse and include genetics and autoimmune disorders, environmental factors, and iatrogenic and idiopathic situations [4, 5]. Current therapeutic management of POF includes psychosocial support, hormone replacement therapy, and fertility management [6]. Infertility treatments for POF focus on oocyte donation. Assisted conception with donated oocytes presently remains the only method of infertility treatment, and it is not accepted by many religions [7, 8].

White et al. detected ovarian stem cells (OSC) in the cortical tissues of ovaries, which enable the generation of oocytes from ovarian tissue in women of reproductive age [9]. In human ovaries, OSC, which is also called the germinal epithelium, is suppressed in fetal midgestation and in adults, it is suppressed during the periovulatory phase. Under these conditions, some OSC are converted into germ cells. Then, the germ cells migrate into the ovarian stroma, and these cells can be activated from the perivascular environment and produce a regenerative microenvironment that secretes bioactive molecules (granulosa cell nest) which aggregates with them and generates primordial follicles [10–12]. Also, it has been shown that in aged mice, the ovarian tissue regenerative microenvironment can restore the capacity of germ cells to make oocytes [13]. Bipotent OSCs exist in POF ovaries [12] that can produce new primordial follicles and epithelial cell nests (primitive granulosa cells); thus, an alternative process, such as autologous stem cell therapy, is a promising treatment [10].

Several studies have reported improved ovarian function and pregnancies in women with POF from chemotherapy and total radiation who had mesenchymal stem cell therapy and bone marrow transplantation of mesenchymal stem cells (MSCs) as part of the their treatment [7, 14, 15].

There are different types of stem cells that can be derived from numerous sources include bone marrow stem cells, placenta MSCs, endometrial MSCs, and adipose-derived stromal cells (ADSCs) [16, 17].

MSCs from different sources have been shown to repair damaged ovarian function in preclinical chemotherapy induced POF animal models [16, 17]. ADSCs, one source of obtain MSCs, are comparable with other stem cells, which makes them suitable for therapeutic purposes and clinical applications. ADSCs can be isolated more easily and in abundant quantities, can be collected by a minimally invasive procedure, and are transplanted safely and efficiently to either an autologous or allogenic host [18]. ADSCs predisposed for oocyte regeneration, increased follicle and oocyte numbers and reduce granulosa cells (GCs) apoptosis in chemotherapy induced POF mice models [16, 19, 20]. However, the principle of MSCs in follicle growth is unknown and its mechanism of action has not been fully elucidated.

Therefore, it is suggested that ADSCs can improve and regenerate the oocyte environment, which would improve fertility results in POF patients. In this study, we performed intraovarian transplants of autologous ADSCs and evaluated their safety, feasibility, efficacy and the dosage adjustment of these ADSCs in POF patients.

## Materials and methods

### Study design and subjects

This non-randomized open label study was approved by the Ethical Committee of Royan Institute and registered at the http://www.Clinicaltrials.gov, (NCT02603744). We designed a guidance pamphlet that presented to couples and especially remarked the risk of neoplasm and followed oophorectomy so that their decision was conversance for transplantation.

After extensive consultation by gynecologists and physicians who specialize in stem cell therapy, the patients fill out informed consent before enrollment then divided into 5, 10 and 15 million received cell groups. Since the transplantation of stem cells in intra-ovarian tissue has not been performed before, ADSCs were transplanted in one ovary for conserves apposite it from any neoplasms and/or abscess.

Nine women had a definite diagnosis of POF based on FSH levels of ≥25 IU/L on two separate occasions within a four-week period; they were 20–39 years of age. As shown Table 1, the inclusion and exclusion criteria are described in detail. All patients had amenorrhea at least 1 year before the study. All participants were following up for more than 1 year from initially diagnosed with POF.

**Table 1:** Patient’s inclusion and exclusion criteria

**Inclusion criteria**	
- 20–39 years of age	
- FSH ≥ 25	
- Normal karyotype	
- Not fragile X mental retardation 1 (*FMR1)* mutations	
**Exclusion criteria**	
- Thyroid disorders	
- Congenital abnormalities of the reproductive tract	
- Liposuction contraindications	
- Autoimmune disorders	
- Previous or any familial histories of ovarian tumors	
- Past history of cancer, chemotherapy, radiotherapy	
- Positive HIV, hepatitis C and B	
- Severe endometriosis	

### Adipose-derived stromal cell (ADSCs) production and flow cytometry

A surgeon aspirated the adipose tissue from sub-abdominal fat pads. The 50 mL adipose tissue sample was placed in a sterile tube that contained phosphate-buffered saline (PBS; Miltenyi Biotech GmbH) and 1% penicillin/streptomycin. The sample was inside a cool box until it was transferred to a clean room. The specimen transferred to Celltech pharmed, a GMP grade company, for cell preparation. The specimens were washed several times with sterile water to remove any hematopoietic contamination. The cell preparation process and quality control tests were based on a previously published protocol [21]. Briefly, the samples were digested in the incubator (5% CO_2_ and 37 °C) using 0.075% collagenase type I enzyme for 2 h. Next, the collagenase enzyme was deactivated by the addition of twice the volume of alpha modified eagle medium (α-MEM, Gibco, Germany), and the samples were centrifuged at 1500 rpm for 5 min to obtain a stromal vascular fraction (SVF). We did not obtain an adequate amount of SVF during the first liposection in one patient (patient number 6 in Table 2) from the 10 × 10^6^ ADSCs group; therefore, the liposuction process was repeated after 6 months.

**Table 2:** Demographic and clinical characteristics of premature ovarian failure (POF) patients

No.	Age	BMI	Cell count	Ovary volume left (cc)	Ovary volume right (cc)	LH (mIU/ml)	FSH 1^**a**^ (mIU/ml)	FSH 2^**a**^ (mIU/ml)	AMH (ng/ml)	AFC	Affected ovary
1	38	27.0	5 × 10^6^	6.6	4.1	72	100.0	100.0	0.10	3	Left
2	30	24.6	5 × 10^6^	1.0	1.5	95	85.0	43.0	0.10	0	Left
3	32	22.3	5 × 10^6^	4.7	4.3	20	25.0	28.0	0.30	2	Right
4	21	21.4	10 × 10^6^	2.9	2	20	41.0	72.0	0.10	0	Right
5	35	29.6	10 × 10^6^	2.1	2	38	52.0	89.0	0.10	0	Left
6	33	21.6	10 × 10^6^	3.0	-^b^	15	59.9	25.0	0.10	0	Left
7	34	26.3	15 × 10^6^	1.3	1	41	64.0	67.0	0.03	0	Right
8	35	24.8	15 × 10^6^	1.0	4	67	86.0	103.0	0.10	3	Right
9	33	21.6	15 × 10^6^	0.75	0.75	17	67.0	98.1	0.20	0	Left

*BMI* Body mass index, *LH* Luteinizing hormone, *FSH* Follicle-stimulating hormone, *AMH* AMH anti-Mullerian hormone, *AFC* Antral Follicle Count

^a^According ESHRE identification FSH evaluated twice;1: Administration FSH; 2: transplantation day FSH

^b^Not detected by vaginal ultrasonography

We used flow cytometry to confirm the purity of the isolated ADSCs between passages passage 0 to passage 3. An adequate amount of cells were examined by flow cytometry for specific surface markers that consisted of positive monoclonal antibodies for CD105, CD90, and CD73 (BD, Pharmingen™, USA) [22].

### Adipose derived stromal cell (ADSCs) administration and transplantation

Prepared autologous ADSCs suspensions of 5 × 10^6^, 10 × 10^6^, or 15 × 10^6^ cells were transplanted into the patients’ ovary. Three patients were assigned to each group of cells. After the cell transplants, each patient was observed for the possibility of early adverse effects.

To ensure that the exact amount of ADSCs was transplanted, the 2 mL cell suspension prepared contained a 1.3 mL accurate amount of either 5 × 10^6^, 10 × 10^6^, or 15 × 10^6^ ADSCs and an additional 700 μl amount that remained in the needle. This value was estimated for a puncture needle (Reproline Medical GmbH, 53,359 Rheinbach, Germany) with an outer diameter of 1.4 mm and 32 cm length. Under general anesthesia; the vagina was washed with normal saline solution and we used a puncture needle under transvaginal ultrasonography (Japan’s Aloka-40,000 vaginal probe) guidance to unilaterally transplant the assigned amount of ADSCs into one of the patient’s ovary. Considering the small size of the ovaries and difficulty to fix and locate these ovaries in two patients, we performed laparoscopy to transplant the ADSCs.

### Objectives and clinical assessments

The primary objective of the current study was to evaluate the safety of ADSCs transplantation. Participants were followed for early-onset possible side effects such as bacteremia, sepsis, PID, anaphylactic shock, and hematoma from the first 24 h and during the first week because of the transplantation procedures. The patients remained in follow up for the second week, and for 1, 2, 3, 6, and 12 months after the transplantation to evaluate safety and effectiveness, and any secondary complications. Possible second side effects such as ovarian abscesses and benign neoplasms were evaluated via physical examination and vaginal ultrasonography. Secondary objectives included and the effects of ADSCs transplantation on the resumption of menstruation, hormones level (FSH and anti-Müllerian hormone [AMH]), ovarian function (AFC and ovary volume by ultrasonography evaluation). Another secondary objective variable was dose escalation. Effectiveness was assessed for the first and second week, and 1, 2, 3, 6, and 12 months after the transplantation. Some patients follow up over 1 year due to returning for their infertility treatment.

### Statistical analysis

We carried out the statistical analysis with IBM SPSS Statistics for Windows, Version 22.0 (IBM Corp., Armonk, NY, USA). In the present study, continuous variables were expressed as mean ± standard deviation/error (SD/SE). Categorical variables were expressed as frequency (percent). One-way ANOVA test was used to assess the effects of treatment by cell count. All statistical tests were 2-sided and *P* < 0.05 was considered statistically significant.

## Results

**Fig. 1:**
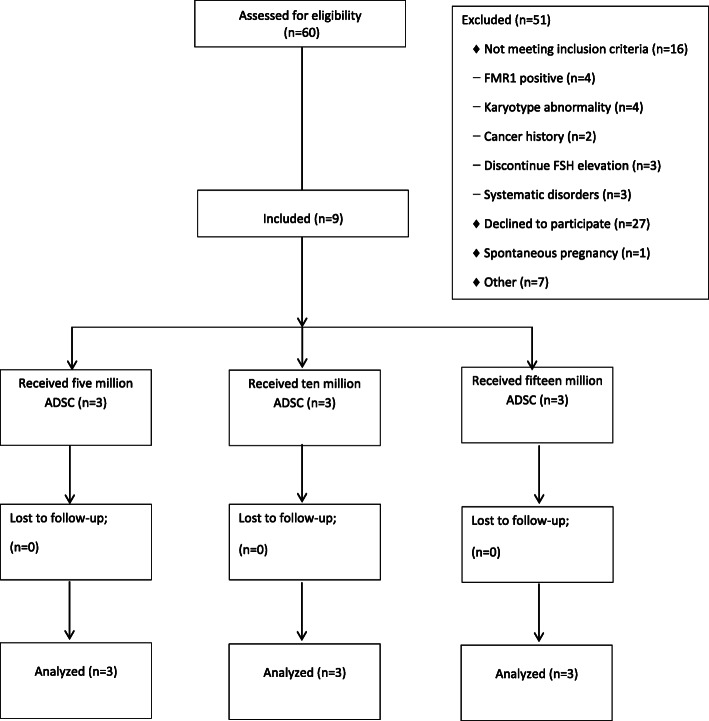
Flow chart showing the disposition of POF participants in this trial

All participants were recruited for participation during 2015 to 2018. Of the 50 women enrolled, nine were completed the study (Fig. 1).

Table 2 lists the demographic and clinical characteristics of the 9 included POF patients. The patients’ average age was 32.2 ± 4.8 years and their average BMI was 24.7 ± 2.88 kg/m^2^. Secondary amenorrhea was observed in 8 (88.9%) patients and one (1.1%) patient had primary amenorrhea (Patient number 4) (Table 2). Comparison of demographic and clinical characteristics by cell count revealed that there were no significant differences (*p* > 0.05).

Participants had not shown any early-onset possible side effects at the first 24 h and during the first week, as well as not detected secondary complications during follow up for the first and second week, and 1, 2, 3, 6, and 12 months after the transplantation by physical examination and vaginal ultrasonography.

The average number of ADSCs was injected in a 2 ml cell suspension with 85–90% viability in all cell count groups (Table 3).

**Table 3:** Average number of cells and immunophenotyping of the autologous adipose derived stem cells

Characteristics/Groups	5 × 10^**6**^ cell count (***N*** = 3) [min-max]	10 × 10^**6**^ cell count (***N*** = 3) [min-max]	15 × 10^**6**^ cell count (***N*** = 3) [min-max]
SVF cell no.	23–30 × 10^6^	21–39 × 10^6^	24–93 × 10^6^
Total injected cell no.	5 × 10^6^	10 × 10^6^	15 × 10^6^
% Viability	97.5–98.0	91.0–98.0	85.0–90.0
% CD90^+^	98.1–99.1	97.3–98.6	98.3–98.5
% CD105^+^	96.1–99.2	92.6–97.9	95.4–96.9
% CD73^+^	87.6–98.5	80.0–90.7	77.2–91.4

*CD* Cluster of differentiation, *no* Number

Table 4 shows the return of menstruation in four patients according to the group in the 9 POF patients. In the 15 × 10^6^ group, two POF patients had a return of menstruation 2 months after the intervention which for one established until the fifth month and another experience menstruation for second and fourth month after cell transplantation. Two other POF patients; one in 5 × 10^6^ and another in 10 × 10^6^ cell group reported menstruation resumption at 4 weeks after the intervention which established for the 8 and 7 months after the intervention, respectively. These differences were not significant between cell groups (*p* > 0.05).

**Table 4:**
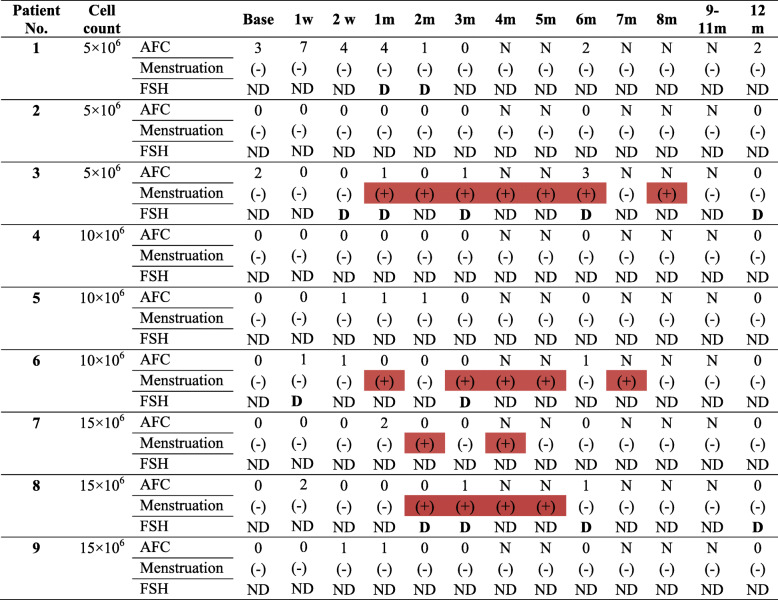
Menstruation and AFC situation in premature ovarian failure (POF) patients at baseline and follow up time

Colored box is indicated for showing return menstruation. Menstruation: positive (+) and negative (−)

*AFC* Antral follicle count, *N* Not applicable, *FSH* Follicle-stimulating hormone, *D* Decline FSH < 25 IU/l, *ND* Not decline < 25 IU/l, *W* Week, *M* Month

**Fig. 2:**
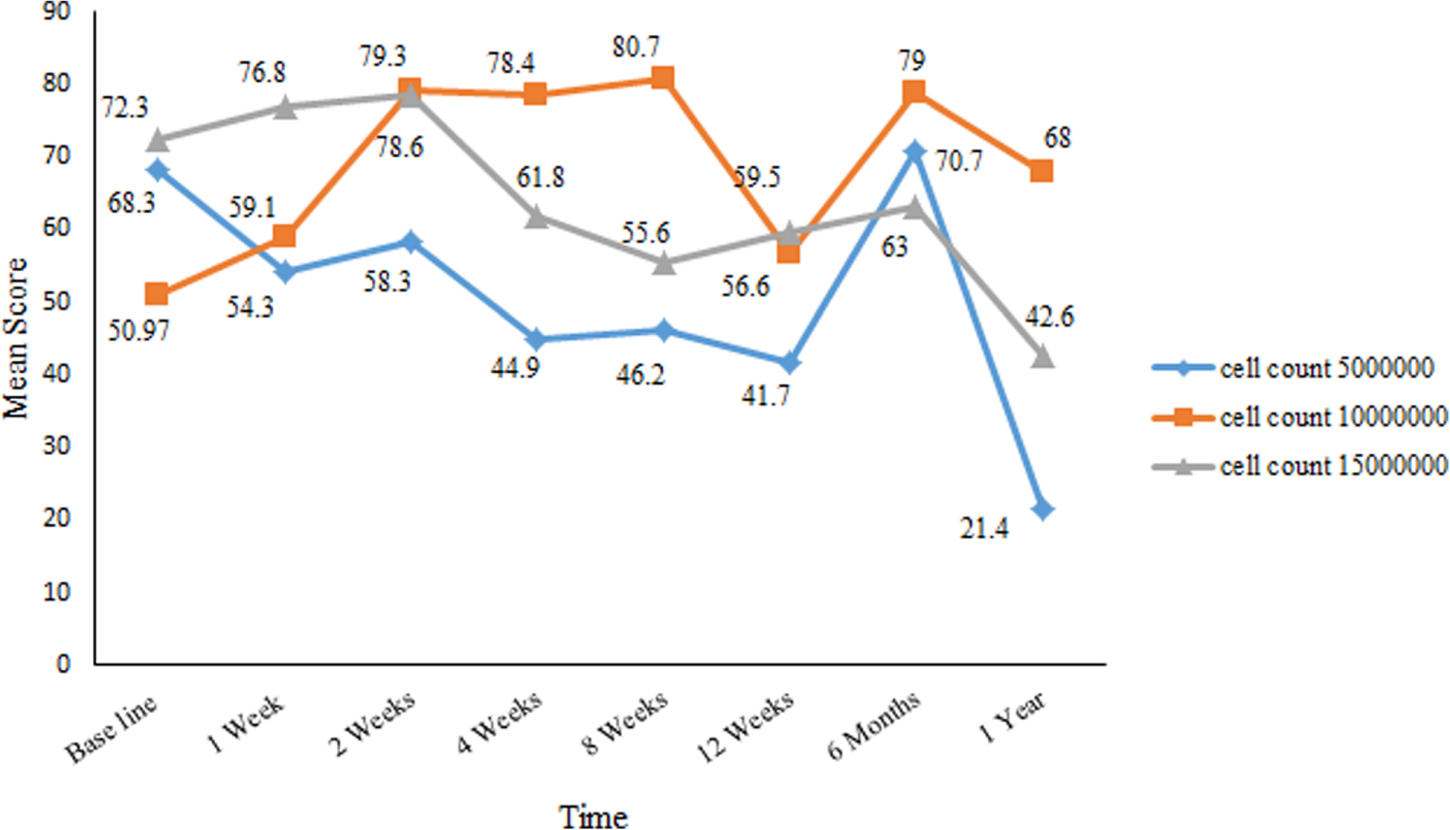
Changes in FSH levels during follow-up in all 9 premature ovarian failure (POF) patients

We observed decreased serum FSH levels less than 25 IU/l in four patients at the weeks 1 and 2 of follow up, the first month, and the second month permanently decreased. In two patients, this decline in FSH level remained stable until 1 year after transplantation. After 5 × 10^6^ ADSCs transplantation, in one patient FSH level decreased in the first (27 IU/l) and second months (18.5 IU/l); however, another patient showed decline FSH in the second week (16 IU/l), first month (21 IU/l), third month (15.4 IU/l), sixth month (7.7 IU/l) and first year (5.9 IU/l) during the follow up. FSH level was declined only in one patient after 10 × 10^6^ ADSCs transplantation at first week (13.5 U/l), second month (24 IU/l) and third months (10.6 IU/l) during follow up. In another patient, who received 15 × 10^6^ ADSCs, the decreased FSH level began from the second month (7.9 IU/l), third month (23.1 IU/l), sixth month (26 IU/l) and first year (4.3 IU/l). In comparison between groups, the FSH level was decline in the 5 × 10^6^ cells group compare to 10 × 10^6^ and 15 × 10^6^ ADSCs groups and the lowest level was observed in 1 year after treatment than baseline in the cell count 5 × 10^6^ group (mean ± SE: 21.45 ± 15.55 vs. 68.3 ± 24.5), *p* = 0.631) (Fig. 2). There were no significant differences in ADSCs transplantation groups in comparison of serum FSH level (*p* > 0.05). Menstrual resumption was observed in 3 of these patients who had decreased FSH levels. One patient in each 5 × 10^6^, 10 × 10^6^ and 15 × 10^6^ ADSCs group who had a sustained decrease in serum FSH reported a return to regular menstruation from the first and second month after transplantation.

**Fig. 3:**
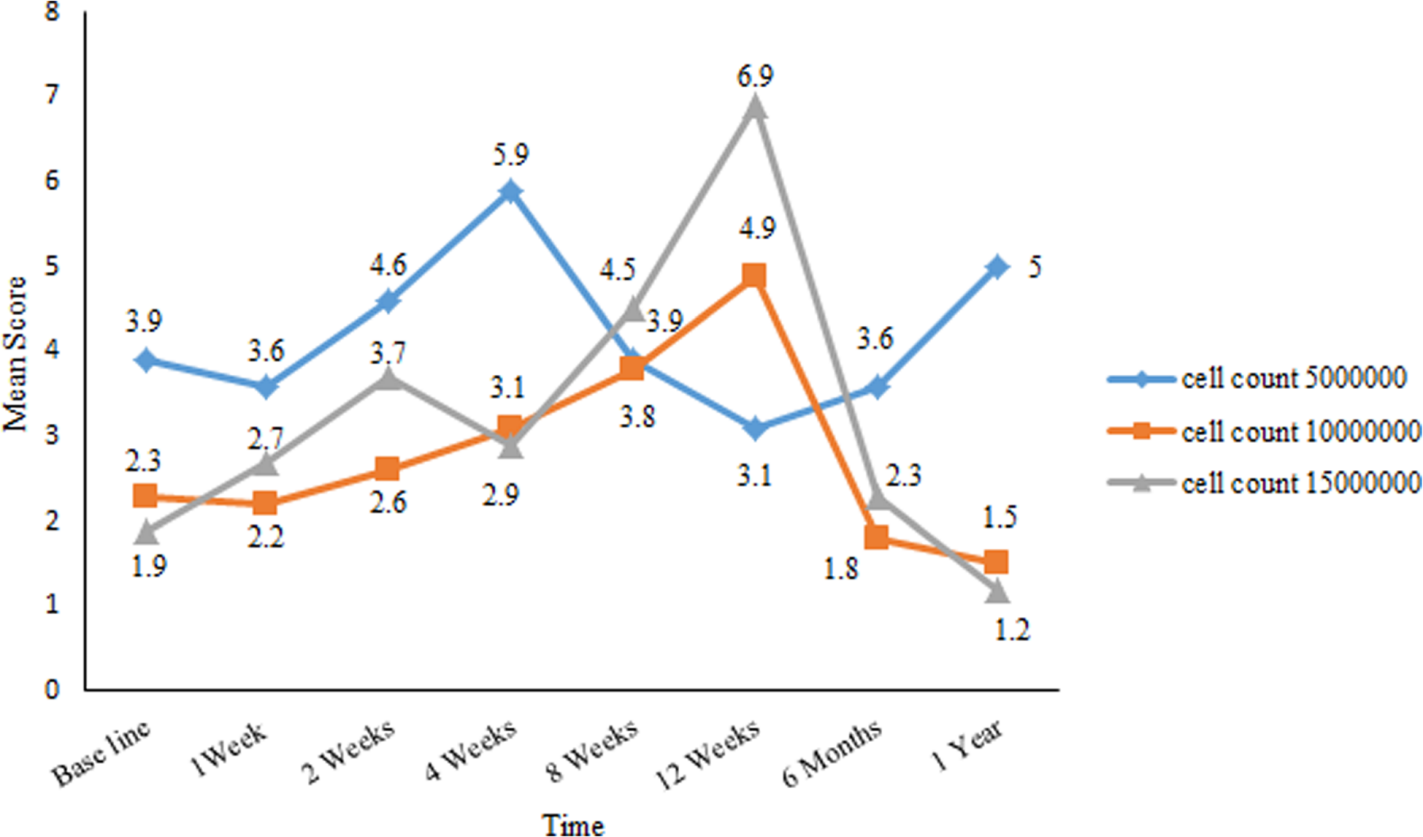
Affected ovary volume changes during the follow-up period in all 9 premature ovarian failure (POF) patients

Figure 3 showed the ovarian volume was mostly variable during follow up; however, statistically significant changes were not seen in ovary volume according to the groups (*p* > 0.05).

**Fig. 4:**
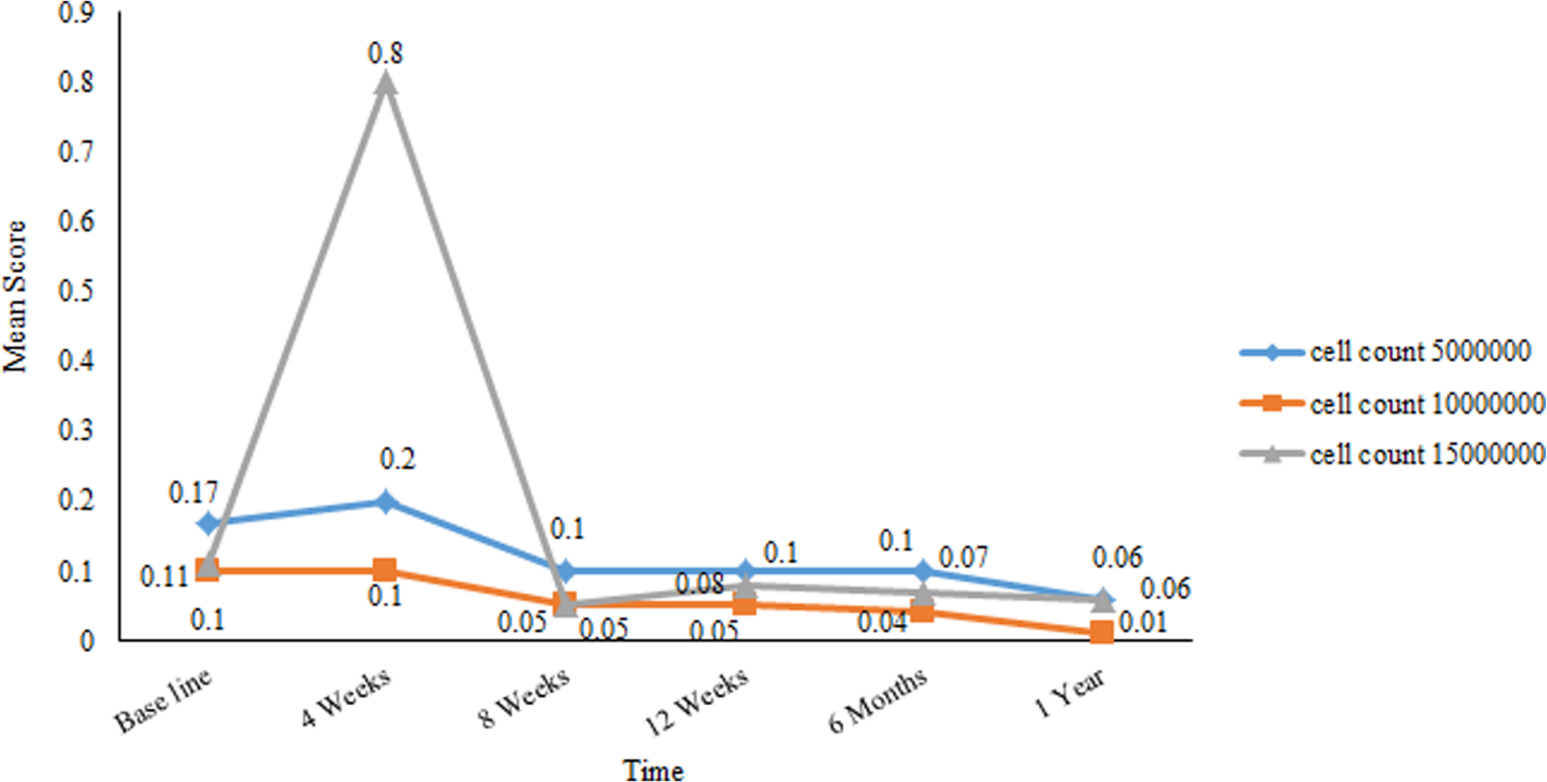
Changes in anti-Müllerian hormone (AMH) level during follow-up in all 9 premature ovarian failure (POF) patients

The AMH serum levels remained almost permanent during the follow up and none of the patients in any of the groups had a stable pattern for increasing or decreasing changes in AMH levels during the follow-up period (Fig. 4). AFC rates did not differ in any of the patients (Table 4). A comparison of serum AMH and AFC revealed that there were no statistically significant differences between ADSCs groups (*p* > 0.05).

## Discussion

Depletion of ovarian primordial follicles, increased follicle loss, or destroyed interaction between oocytes and granulosa cells are causes of POF, but the ovarian function could be reversed by stem cell therapy [10–12]. This is particularly true in cases of idiopathic POF, which comprises approximately 90% of POF cases [23].

This current study, a non-randomized phase I clinical trial, was designed to assess the safety, feasibility, effectiveness and dosage adjustment of three doses (5, 10, and 15 × 10^6^) of autologous ADSCs administered via unilateral intraovarian vaginal or laparoscopy transplant in 9 patients with idiopathic POF who met the diagnostic criteria for POF.

Most studies described the efficacy of MSCs in women who underwent chemotherapy, irradiation, and bone marrow allograft transplantation especially in rodent POF models [15]; however, recently restricted human clinical trial in this area was reported.

Edessy et al. reported the first autologous bone marrow stem cell transplantation via laparoscopy into the ovaries as treatment for idiopathic POF. The results showed recovered menstruation in two cases (20%) 3 months after the transplantation. One case (10%) became pregnant and delivered the first baby from this therapeutic procedure [24].

Similar to the results reported by Edessy, in this study, we reported that all patients with idiopathic POF had amenorrhea before treatment; of these, four patients resumed menstruation. Two of the four experienced menstruation 1 month after the transplantations, and two patients experienced it in the second month, which continued for 3, 5, 7 and 8 months after the transplantation.

Gupta et al. reported the case of a 45-year-old perimenopausal women who underwent intraovarian transplantation of autologous bone marrow stem cells by laparoscopy. After 8 weeks, her AMH level increased from 0.4 ng/mL to 0.9 ng/mL. An ultrasound imaging identified two follicles in each ovary and the patient became pregnant after a frozen embryo transfer cycle, which resulted in a live birth [25].

In our study, the AMH level and AFC did not increase; three patients have similar AFC before and after ADSCs transplantation and two of patients show AFC after transplantation. Both AMH and AFC could be used as markers to evaluate ovarian reserve generally or in general population [26, 27] which is not important for POF definition.

AMH is produced by the preantral follicles and it shows a positive correlation with AFC [28]. However, it should be considered that increased AMH levels more observed in patients with a higher number of follicles compared to few ovarian follicles [28] thus minute changes in AMH levels have no significant effect on ovulation response and could not be an indication of oocyte revival. Although altered in several tests were detected in POF, FSH level is a valuable marker and better predictive indication for the ovarian response which definitely according to ESHRE guideline [3]. Because POF patients have not menstrual cycle, their FSH level is highly variable and we considered cut of FSH level > 25 which proposed of ESHRE. In our study, for patients with menstrual resumption, FSH levels measured at 2–3 days of the menstrual cycle.

There is no clinical trial of ADSCs therapy for POF. ADSCs have only been investigated in an animal model. In this model, Sun et al. performed bilateral intravenous transplantations of ADSCs in mice as a chemotherapy-induced POF model. They concluded that improved ovarian function increased the numbers of follicles and induced ovulation. They observed that transplantation of ADSCs enhanced the production of vascular endothelial growth factor (VEGF), placental growth factor (PGF), and transforming growth factor-β (TGF-β), which are involved in oogenesis [29].

Although the cited study is an animal model investigation, in the current study, we evaluated the ovary volume, AFC, and AMH that not shown significant difference. However, serum levels of FSH were consistently reduced in four patients that remained stable until the end of follow up for two patients (third and eighth patients) in 5 and 15 million cell count groups who resumed menstruation. Two of the four patients that presented resumption of menstruation had the prolonged lowest baseline FSH (< 25 IU/l).

Menstrual resumption and decline FSH level are positive findings following ADSCs transplantation which not significantly different between groups which suggest that response to ADSCs treatment is independent of the cell count.

The mechanism by which stem cell affect follicular renewal is not clear; however, the evidence explains neo-oogenesis in adult ovaries. Follicular renewal in ovaries results from bipotent OSC division in the tunica albuginea layer of the ovary, and has the capacity to differentiate into primitive granulosa cell nests and germ cells. Of note, the formation of new adult primordial follicles arises through the granulosa cell nest [10–12, 30, 31]. Xia et al. has shown that a co-culture of MSCs with follicles extracted from the human ovary promotes the growth and development of antral follicles, which represents modified microenvironment for the oocytes [32].

Transplanted stem cells restore ovarian function due to cellular and other local signaling, including immune system-related activated OSCs, and salvaging the sufficient number of existing oocytes as well as repairing damaged ovarian niches [10–12].

As mentioned above, there are few studies on ADSCs therapy that propose ADSCs have limited differentiation potential and do not develop into follicles; therefore, follicular restoration occurs via improving damaged ovarian niches by altered cytokine and hormone levels [16].

Taken together, in the current study, we followed the patients for 12 months to assess the safety of the ADSCs transplantations. During and at the end of the study, local or systemic side effects were not observed according to physical examinations, and laboratory and ultrasonography assessments. Although no clinical trial has assessed the impact of ADSCs on POF, several studies have demonstrated that local and systemic transplantation of ADSCs in women who underwent chemotherapy are safe and without complications [14, 15, 33]. The results of the current study confirm the findings of previous investigations of POF treatments.

## Conclusion

This study showed that clinical applications of ADSCs therapy are safe more than 1 year and feasible and efficacy (decrease FSH and resumption menstrual). The results for each cell count were not significantly different, and some variables showed favorable results after transplantation with the 5 × 10^6^ dose of ADSCs. We suggest that to achieve the best results, the 5 × 10^6^ ADSCs can be transplanted two times and bilaterally into the ovaries because they provide a rich microenvironment for both ovaries as well as had cost-benefit. However, further studies with larger numbers of patient in different clinical trial stage would be needed.
